# Exploiting Spiropyran Solvatochromism for Heavy Metal Ion Detection in Aqueous Solutions

**DOI:** 10.1021/acsomega.5c04821

**Published:** 2025-08-06

**Authors:** Nathália E. N. Mendonça, Carlos A. S. Leão, Frank Alexis, Valeria Ochoa-Herrera, Aracely Zambrano-Romero, Roberto S. Nobuyasu, Flávio B. Miguez, Frederico B. De Sousa

**Affiliations:** a Laboratório de Sistemas Poliméricos e Supramoleculares (LSPS) - Instituto de Física e Química, Universidade Federal de Itajubá (UNIFEI), Itajubá, MG 37500-903, Brazil; b Laboratório de Fotofísica Molecular - Instituto de Física e Química, Universidade Federal de Itajubá (UNIFEI), Itajubá, MG 37500-903, Brazil; c Departamento de Ingeniería Química, Institute of Energies and Materials − Colegio de Ciencias e Ingenierías, Universidad San Francisco de Quito (USFQ), Quito 170901, Ecuador; d Colegio de Ciencias e Ingenierías, AQUA-BIO LAB, Universidad San Francisco de Quito (USFQ), Quito 170901, Ecuador

## Abstract

Three spiropyran derivatives (**SP1**–**SP3**) were studied as colorimetric sensors for Cd^2+^, Pb^2+^, and Hg^2+^ ions in nine solvents of varying polarity and hydrogen-bonding character based on the E_T_(30) scale. We used solvatochromic analysis and UV–visible titrations to characterize each spiropyran’s baseline absorption in each solvent and its changes upon metal interaction. Solvatochromic measurements enabled us to separate intrinsic solvent-induced color shifts from metal-specific responses. The results revealed a strong solvent dependence for sensing. **SP1** and **SP2** yielded distinct metal-induced colorimetric responses, with the former showing its strongest Hg^2+^ response in tetrahydrofuran and acetone and the latter detecting Pb^2+^ and Cd^2+^ in methanol, whereas **SP3** was comparatively less sensitive overall. Notably, Hg^2+^ elicited the most selective and sensitive colorimetric response (inducing a purple to yellow transition), Cd^2+^ presented clear blue shifts (with a color change from purple to dark pink and pink to colorless), and Pb^2+^ caused only minor spectral changes in one system. These findings highlight that solvent polarity can be utilized to modulate spiropyran–merocyanine equilibria and interaction affinities. Selectivity tests in real contaminated water samples confirmed that the **SP1** probe retains high specificity for Hg^2+^ even in the presence of common interfering ions. Overall, this study highlights the potential of solvent-tuned spiropyran probes for rapid, selective detection of toxic heavy metals (particularly Hg^2+^) in environmental monitoring applications.

## Graphical abstract

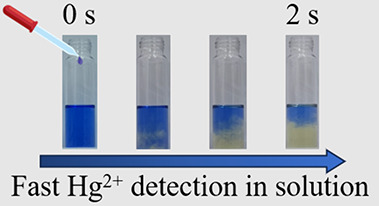

## 1 Introduction

Heavy metal contamination is one of the most pressing challenges we face today, affecting both our environment and public health due to the persistent, bioaccumulative, and nature of heavy metals such as Hg^2+^, Pb^2+^, Cd^2+^, and others.^1,2^ For example, mercury, particularly when converted into its organic form as methylmercury, tends to bioaccumulate in aquatic food webs, leading to high concentrations in fish that can subsequently affect human consumers.^3^ Lead is well-known for its neurotoxic effects; even low-level exposure, especially in children, can impair cognitive development and lead to behavioral problems.^4^ Meanwhile, cadmium is associated with kidney and liver damage and has been classified as a potential human carcinogen.^5^

These heavy metals can enter the environment through natural processes, such as weathering of metal-rich rocks and volcanic activity, but anthropogenic sources are of greater concern.^6,7^ Industrial discharges,^8,9^ mining operations,^10,11^ improper waste management,^12^ and agricultural practices^13^ have significantly increased the levels of these metals in water, soil, and air. Studies have documented that even minimal concentrations of heavy metals can disrupt biological processes, leading to adverse effects on ecosystems and human health.^14,15^

Conventional analytical techniques provide excellent sensitivity and selectivity for heavy metal detection; however, their high cost, complex instrumentation, and time-consuming sample preparation procedures limit their practical application in field monitoring and rapid diagnostics. Consequently, there is a current need for alternative approaches that are not only sensitive and selective but also cost-effective, portable, and amenable to real-time monitoring.^16,17^ In this context, optical chemosensors, particularly those based on spiropyran derivatives, have emerged as promising candidates for heavy metal ion detection.^18–20^

Spiropyrans constitute a well-studied class of photochromic molecules that exhibit a reversible transformation between a closed, colorless spiro (SP) form and an open, colored merocyanine (MC) form upon stimulation by various external factors such as UV/visible light, pH changes, or specific chemical interactions.^21,22^ This transformation involves a significant change in molecular conjugation and dipole moment, which is readily observable as a color change and/or a modulation in fluorescence properties.^23,24^ Such characteristics make spiropyrans strong candidates for sensor applications where an optical response is used to indicate the presence of a target analyte.^25,26^

The sensitivity of spiropyran-based sensors arises from the environmental responsiveness of the SP-MC equilibrium. In the presence of certain metal ions, the coordination interactions between the metal and the donor sites (typically oxygen or nitrogen atoms) within the spiropyran molecule can shift the equilibrium toward the colored MC form.^27,28^ This ion-induced stabilization results in a distinct and rapid colorimetric response that can be easily detected by the naked eye or through spectroscopic means.^21^ In a recent study, researchers developed a dual-chromic, cellulose-based sensor.^29^ This sensor was fabricated by modifying ordinary cellulose paper with polyacrylic nanocapsules containing a combination of a thermochromic leucodye and a spiropyran derivative. The material undergoes rapid and distinct color changes when exposed to Fe^2+^, Sn^2+^, and Pb^2+^, with limits of detection (LODs) significantly lower than the thresholds established by the World Health Organization.

Recent advancements in molecular design have allowed researchers to tailor the spiropyran scaffold to enhance binding affinity and selectivity toward specific metal ions.^30–32^ Structural modifications, such as the introduction of electron-donating or -withdrawing substituents, have been shown to significantly influence the optical properties and metal ion recognition capabilities of spiropyran derivatives.^21^ For instance, by strategically placing functional groups that act as additional coordination sites, we can markedly improve the sensitivity of spiropyran sensors toward heavy metals. These modifications not only facilitate stronger metal–ligand interactions but also enable a more pronounced modulation of the SP-MC equilibrium upon analyte binding.^33–35^ Such improvements are particularly critical when targeting specific metals, which are of high environmental concern due to their extreme toxicity even at low concentrations.

Despite these promising developments, several challenges remain. One of the primary concerns is a common problem with chemosensors, which is the potential for interference from competing ions present in complex environmental samples, which can compromise both the sensitivity and the selectivity of spiropyran-based sensors. In this regard, the solvatochromic properties of spiropyrans offer a promising strategy to mitigate these issues. Because solvatochromism is highly sensitive to the polarity of the surrounding medium,^36^ it provides an additional layer of discrimination between different metal ions based on their solvation environments and coordination effects. By integrating solvatochromic behavior into sensor design, it is possible to enhance the specificity of metal ion detection by differentiating analytes not only by their direct interaction with spiropyran but also by their influence on the microenvironment.

Herein, three spiropyran derivatives (Figure 1) were obtained and their solvatochromic properties were studied in nine different organic solvents with distinct dipole moments and hydrogen bonding capabilities.^37^ Taking advantage of the solvatochromic properties of these derivatives, a study was conducted to assess their ability to selectively detect three of the most common heavy metal ions, cadmium, lead, and mercury, by means of spectroscopy analysis and their visual detection by colorimetric changes. This strategy has the intrinsic advantage of excluding further chemical modifications and integration with material devices.

**Figure 1:**
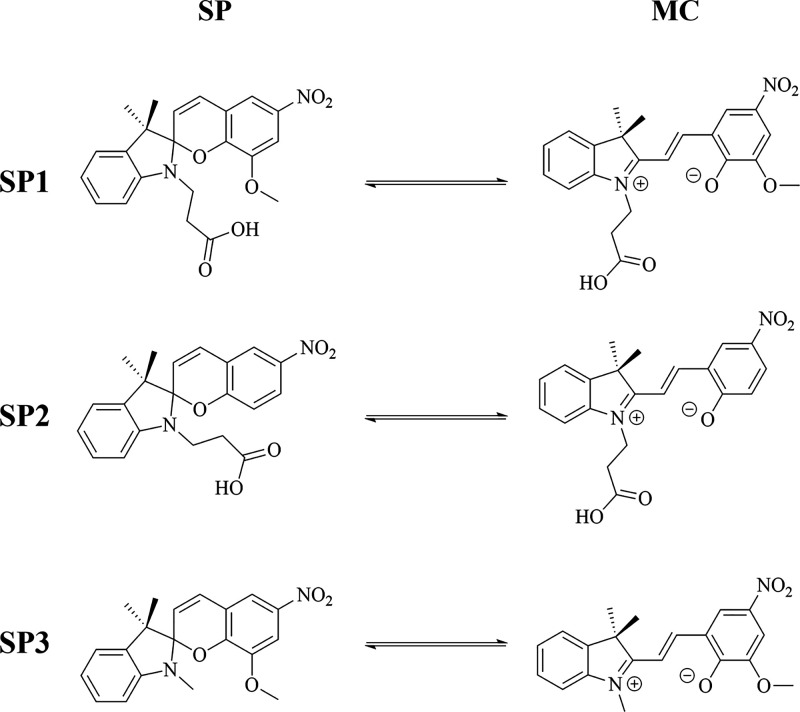
Isomer forms of spiropyran derivatives used in this study: the closed form (SP) and their open-ring form (MC).

## 2 Materials and Methods

### 2.1 Reagents and Solvents

Chemicals were purchased from commercial sources and used without further purification. The following reagents were acquired from Sigma-Aldrich: 2,3,3-trimethylindolenine (98%), 1,3,3-trimethyl-2-methyleneindoline (97%), 3-iodopropanoic acid (95%), 2-hydroxy-5-nitrobenzaldehyde (98%), 3-methoxy-5-nitrosalicylaldehyde (98%), 4-methylpiperidine (96%), methanol (MeOH) (≥99.8%), 2-butanone (≥99.0%), and diethyl ether (Et_2_O) anhydrous (≥99.0%). Acetonitrile (MeCN) (≥99.5%) and acetone (Ace) (≥99.5%) were from Êxodo Científica. Dichloromethane (CH_2_Cl_2_) (>99.5%), 1-propanol (PrOH) (≥99.5%), 1-butanol (BuOH) (≥99.5%), and 1-octanol (OcOH) (≥99.5%) were from Quimex. Ethanol (EtOH) (≥99.8%) was from Honeywell. Isopropyl alcohol (IPA) (≥99.9%) was from LS Chemical. Tetrahydrofuran (THF) (≥99.9%), cadmium­(II) nitrate (Cd­(NO_3_)_2_·4H_2_O) (>95.5%), and lead­(II) nitrate (PbNO_3_)_2_ (>95.5%) were from Dinâmica. Mercury­(II) nitrate monohydrate (Hg­(NO_3_)_2_·H_2_O) (>98.0%) was from Vetec.

Spiropyran derivates in this work were prepared according to previously published literature procedures (Supporting Information - SI 1). Detailed structural characterization including ^1^H and ^13^C NMR, Q-TOF mass spectrometry, and Raman spectroscopy can also be found in our previous work.^25^

### 2.2 UV–Visible Analysis of **SP1**, **SP2**, and **SP3** Spiropyrans

All UV–visible absorption spectra were obtained at 25 ± 1 °C. Spectra were measured in the UV–visible range (from 200 to 800 nm) using a Varian Cary 50 Scan spectrophotometer and quartz cuvettes with a path length of 10 mm and 1.5 mL. For all UV–vis analyses, spiropyran derivatives were prepared as 0.025 mmol L^–1^ solutions in one of the following organic solvents: THF, Ace, MeCN, OcOH, IPA, BuOH, PrOH, EtOH, or MeOH. Solvatochromic studies included an irradiation of the solutions with a 365 nm diode excitation.

Heavy metal stock solutions were prepared in each of the respective solvents mentioned above at 1.2 mmol L^–1^ for the screening experiment. A single 10 μL aliquot was added to 1 mL of the spiropyran solution, and the corresponding absorption spectrum was recorded. For the titration experiments, stock solutions were prepared at 0.24 mmol L^–1^ for Cd^2+^ and Pb^2+^ and 9.3 μmol L^–1^ for Hg^2+^ in Milli-Q water. A total of 10 sequential additions of 10 μL each were made to 1 mL of the spiropyran solution with an absorption spectrum recorded after each addition.

### 2.3 Steady State Emission Analysis of **SP1**, **SP2**, and **SP3** Spiropyrans

Solutions were prepared with spiropyrans at a concentration of 0.01 mmol L^–1^ with THF, Ace, MeCN, OcOH, IPA, BuOH, PrOH, EtOH, or MeOH. Photoluminescence experiments were performed in solution with quartz cuvettes with a path length of 10 mm and 1.5 mL, excited with a commercial diode laser λ_exc_ = 405 or a diode λ_exc_ = 365 nm, and the spectra were collected via an optical fiber connected to an Ocean Optics USB2000 compact spectrometer.

### 2.4 Metal Analysis in Real Samples

Metal analysis in two real water samples was conducted based on prior research.^38^ Briefly, metal analysis of filtered and acidified water samples was performed using an ICP-OES (Thermo Scientific iCAP 7400) following SM 3120B. Calibration utilized a multielement standard (100 mg·L^–1^), and quality control included certified reference material (ERA 500 WatR). Recovery percentages (90.10–106.27%) were used to adjust concentrations and ensure accuracy.

## 3 Results and Discussion

### 3.1 Solvatochromism

Solvatochromism is a phenomenon typically observed in organic molecules with an extensive π-conjugated electron system, where solvent-molecule interactions induce shifts in the electronic absorption spectrum. These spectral changes arise from variations in solvent polarity, hydrogen bonding, and dispersion forces, which influence the electronic distribution within the molecule.^39^ Among the various solvent polarity parameters, Reichardt’s empirical parameter (E_T_(30)) is one of the most comprehensive and widely used, as it accounts for multiple solvent characteristics.^37^ This parameter will be employed in the analysis described below.

Solvatochromism experiments were performed with UV–vis absorption spectroscopy for the three derivatives **SP1**, **SP2**, and **SP3** in eight organic solvents: acetone, acetonitrile, tetrahydrofuran, methanol, ethanol, 1-propanol, isopropyl alcohol, 1-butanol, and 1-octanol (Figure 2a–c). The interaction between the solvent and solute, known as solvation, is a stabilizing process in which solvent molecules adjust their positions and orientations to minimize the overall interaction energy. In the visible region, MC absorbs light from its π-electron transitions, with the maximum absorption wavelength reflecting the average energy gap between its ground and excited states. Solute–solvent interactions affect the energy levels of these states differently based on their charge distributions.

**Figure 2:**
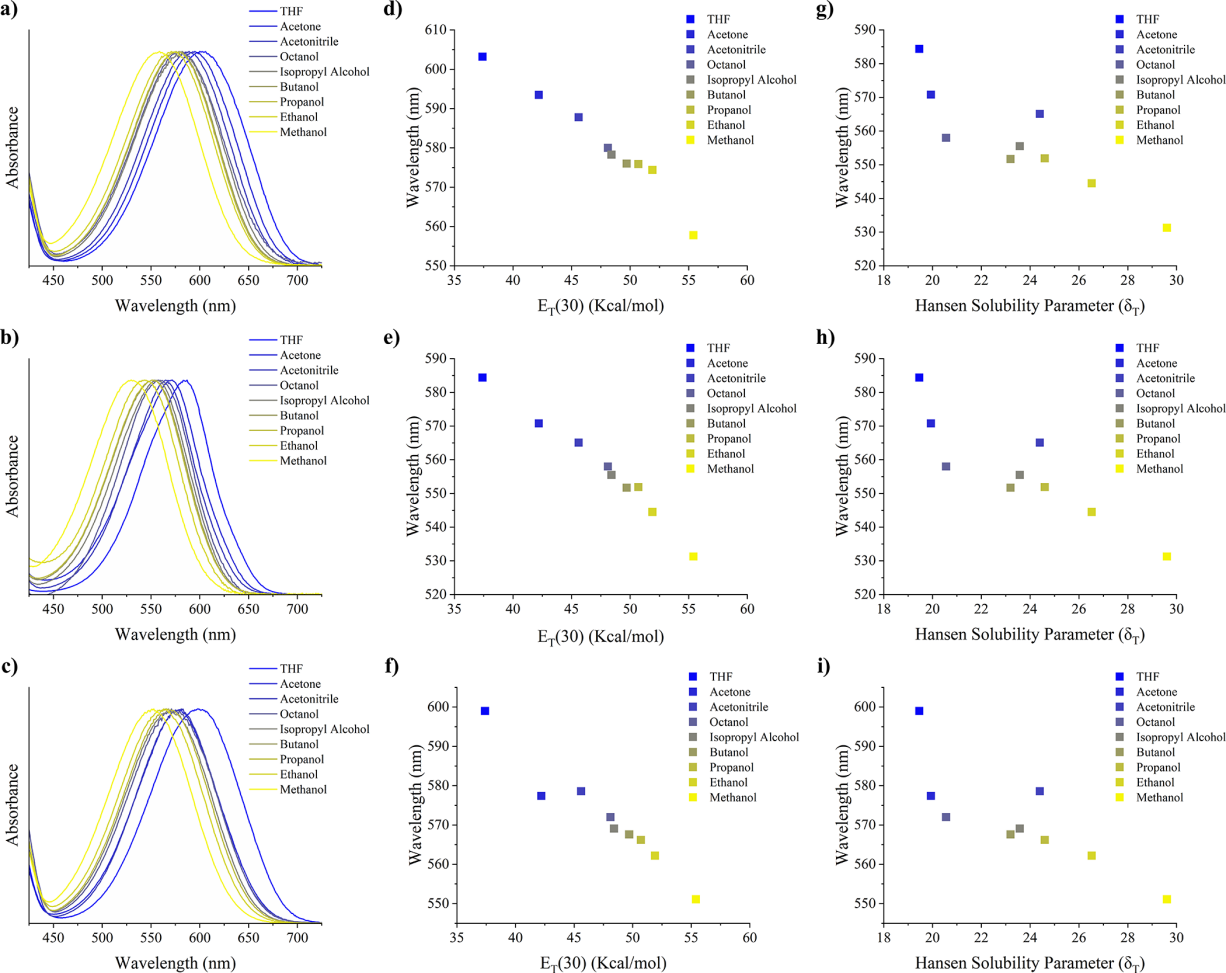
UV–vis spectra in 9 different solvents with (a) **SP1**, (b) **SP2**, and (c) **SP3**. Relationships between λ_max_ of the absorption bands in the visible region and E_T_(30) parameter with (d) **SP1**, (e) **SP2**, and (f) **SP3** and Hansen solubility parameter δ_T_ with (g) **SP1**, (h) **SP2**, and (i) **SP3**.

The maximum absorption wavelength for each derivative varied within the following ranges: **SP1** (559–603 nm), **SP2** (530–587 nm), and **SP3** (553–598 nm). In all cases, the highest wavelength was observed in THF, while the lowest occurred in methanol. This trend aligns with previously reported solvatochromic behavior of similar spiropyran derivatives in the literature.^40,41^

Notably, all three derivatives exhibit negative solvatochromism, meaning that as the solvent polarity increases, the wavelength of the absorption band in the visible region decreases. This trend is illustrated in Figure 2d–f, where the wavelength is plotted against the E_T_(30) parameter. The overall behavior of the spiropyrans demonstrates a strong linear correlation, with *R* ^2^ values of 0.96 for **SP1**, 0.97 for **SP2**, and 0.94 for **SP3**, indicating that the observed spectral shifts are primarily influenced by the solvent polarity. While other solvent properties, such as hydrogen bonding and dispersion forces, may also contribute to these shifts, they were not fully accounted for in this analysis.

Another parameter commonly used in solvatochromism studies is the Hansen solubility parameter (δ_T_), which accounts for dispersion forces, polarity, and hydrogen bonding capacity. In our analysis, we plotted the same maximum absorption wavelength data against δ_T_ (Figure 2g–i) to determine whether a similar linear relationship would emerge. However, we observed a significant loss in linearity compared to the correlation with the E_T_(30) parameter for all spiropyran derivatives, suggesting that δ_T_ may not be as effective in describing the solvatochromic behavior of these molecules.

The fluorescence emission spectra (Figure 3) indicate that the red emissions are influenced by the solvent environment, similar to the trends observed in the absorption spectra. Notably, the Stokes shifts are quite large, ranging from 91 to 113 nm for **SP1**, from 76 to 135 nm for **SP2**, and from 72 to 118 nm for **SP3** (from THF to MeOH). For both **SP1** and **SP3**, the maximum absorption wavelengths measured in protic solvents (the six alcohols) are quite similar, differing by only about 4 nm, within the margin of instrumental error. This similarity may be attributed to the presence of a methoxy group in the benzopyran moiety of both compounds. In contrast, for the three aprotic solvents (THF, Ace, and MeCN), **SP1** exhibits absorption at higher wavelengths. This shift is likely due to the carboxylic acid group in its indole moiety, which can form hydrogen bonds with aprotic solvents, thereby reducing the energy gap for both absorption and emission. In the case of **SP2**, the absence of the methoxy group may destabilize the benzopyran ring relative to those of **SP1** and **SP3**, resulting in lower wavelengths for both its emission and absorption bands. No significant difference was found for **SP2** using a different excitation wavelength (365 nm - Figure 3d), while the other two derivatives did not present relevant emission with this excitation wavelength.

**Figure 3:**
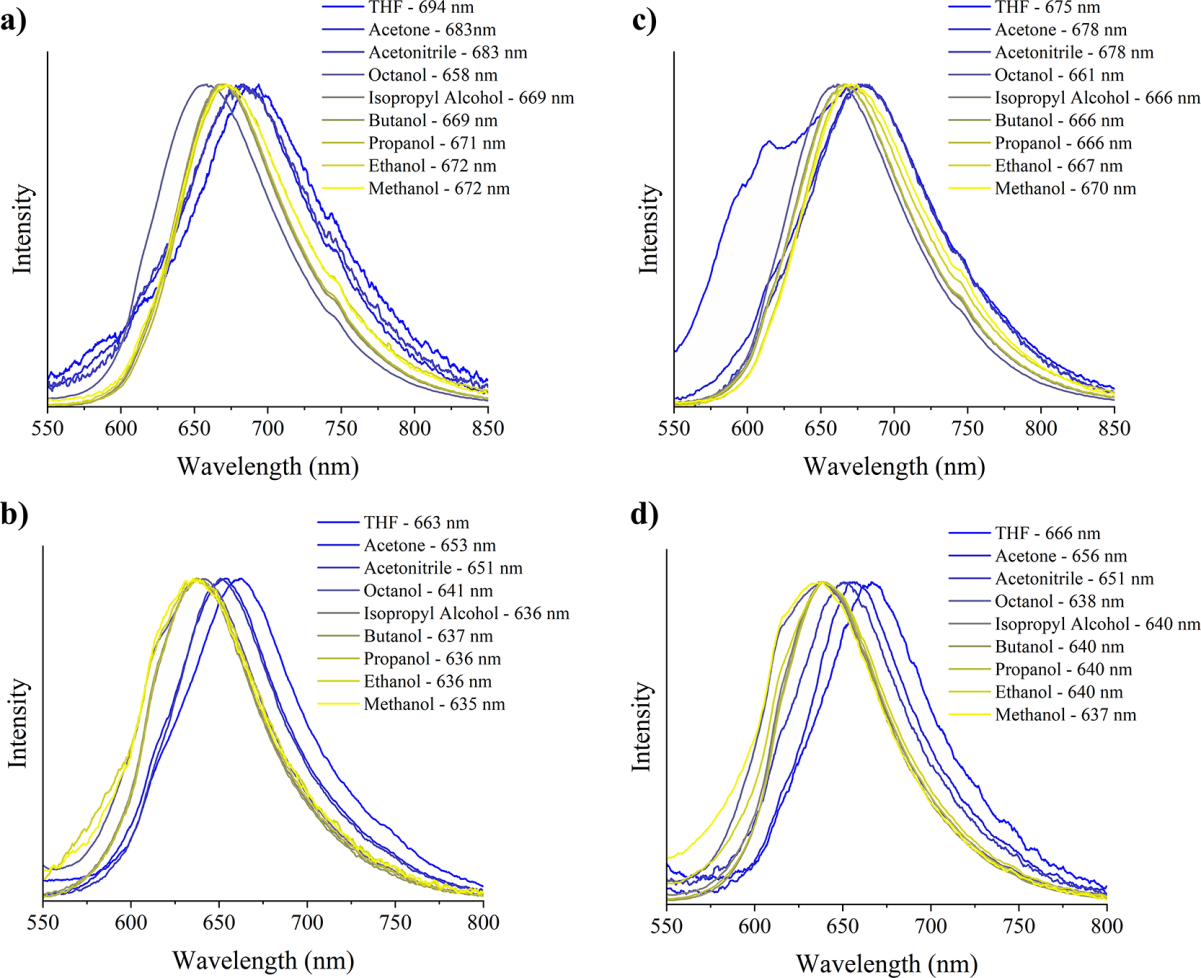
Emission spectra in 9 different solvents using a 405 nm diode laser excitation of (a) **SP1**, (b) **SP2**, and (c) **SP3** and (d) using a 365 nm diode excitation of **SP2**.

### 3.2 Heavy Metal Screening

To investigate the interactions between spiropyran derivatives and heavy metal ions in different solvent environments, a preliminary screening experiment was conducted. This experiment aimed to assess the initial response of three spiropyran derivatives (**SP1**, **SP2**, and **SP3**) to the presence of three heavy metal ions (cadmium, lead, and mercury) across a set of eight solvents with varying polarity and hydrogen bonding capabilities (THF, Ace, MeCN, IPA, BuOH, PrOH, EtOH, and MeOH). OcOH was excluded from the study going forward due to the spiropyrans’ solubility issues, which might have affected quantitative measures. This initial screening provided valuable insights into which spiropyran-metal-solvent combinations exhibited the most significant spectral responses, guiding further titration experiments to quantify binding affinities and colorimetric changes.

After the addition of each heavy metal solution to the spiropyran solution, the final concentration reached 0.012 mmol·L^–1^, exceeding the maximum recommended levels set by the World Health Organization for these metals. However, this concentration was deemed appropriate for the initial assessment, as it allowed for clear observation of both colorimetric and spectral changes. All 72 spectra are presented in Figure SI 2.

Shifts in the maximum absorption wavelength of greater than 15 nm or a near-complete quenching of the visible band were the primary spectral features analyzed. As anticipated from previous studies and literature reports, most spiropyran-metal-solvent combinations did not induce significant spectral or colorimetric changes. Notably, **SP3** was inactive for all combinations of heavy metals and solvents. The lack of interaction (in solution) between the **SP3** and the tested heavy metal ions may be due the spiropyran structural limitation, particularly the absence of strongly coordinating functional groups in the indoline nitrogen, as presented in the **SP1** and **SP2** derivatives. Other researchers have also reported that, in addition to the phenolic oxygen, the presence of a carboxyl acid moiety is important to interact with ions in solution.^42^ **SP1** showed no response to Pb^2+^ in any solvent but successfully detected Cd^2+^ in Ace, IPA, PrOH, and MeOH, as well as Hg^2+^ in THF, Ace, and EtOH. Meanwhile, **SP2** identified Cd^2+^ in Ace and MeOH, Pb^2+^ in IPA and MeOH, and Hg^2+^ in Ace, MeCN, and EtOH.

The results from this preliminary screening highlight how different spiropyran derivatives exhibit selective sensitivity to specific metal ions depending on the surrounding solvent environment. For instance, **SP1** demonstrated a strong response to Cd^2+^ and Hg^2+^, while **SP2** showed broader sensitivity across multiple solvents, particularly for Hg^2+^. **SP3**, in contrast, exhibited a minimal response, with a notable change only in Ace for Pb^2+^. These findings suggest that the solvatochromic behavior of spiropyrans, influenced by solvent properties, plays a crucial role in modulating their binding interactions with metal ions and also guides the titration studies to better understand the quantitative aspect of metal detection.

### 3.3 Quantitative Analysis of Heavy Metal Detection

Based on the results from the screening experiment, only 15 combinations of spiropyrans, heavy metal ions, and solvents demonstrated promising spectral changes indicative of potential for further investigation. In addition to the general influence of solvent polarity on the absorption wavelength of each spiropyran, the water content in the solvent mixtures also plays a critical role in their solvatochromic behavior.^43^ To account for the fact that the heavy metal ions were solubilized in Milli-Q water, control titrations using pure water were performed alongside each metal titration. These controls allowed us to determine whether the observed spectral changes were genuinely due to the presence of the metal ions or simply a result of increased water content in the solution. All control titrations are presented in Figure SI 3.

By comparing the metal ion titrations with the corresponding water controls, only six of the initial spiropyran-metal-solvent combinations showed a significant shift in the visible absorption band or a pronounced decrease in absorbance that could not be attributed to the water content in the solutions; these titrations are presented in Figure 4. For **SP1**, promising results were observed with cadmium (Figure 4a,b) in Ace and PrOH and mercury (Figure 4c,d) in acetone and THF. For **SP2**, both cadmium and lead in methanol (Figure 4e,f) produced spectral changes indicative of an interaction with the spiropyran. In contrast, **SP3** did not show any reliable response to the selected heavy metals; all spectral variations observed during its titrations were comparable to those seen in the water control experiments, suggesting that the changes were likely due to the water content rather than metal binding.

**Figure 4:**
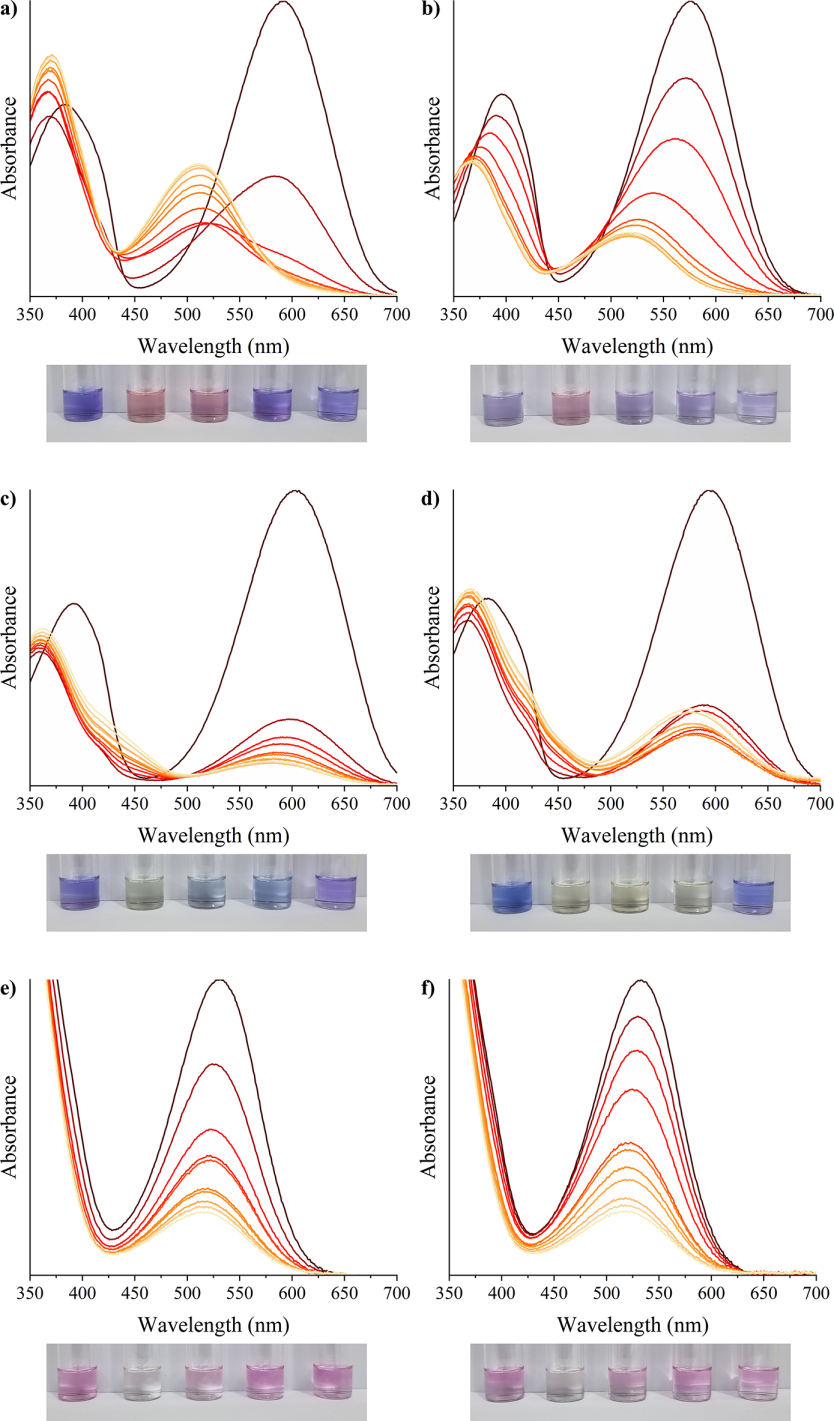
Absorption spectra from titrations with ten successive 10 μL additions of heavy metal stock solutions (0.24 mmol·L^–1^ for Cd^2+^ and Pb^2+^; 9.3 μmol·L^–1^ for Hg^2+^) for (a) **SP1** in Ace using Cd^2+^, (b) **SP1** in PrOH using Cd^2+^, (c) **SP1** in THF using Hg^2+^, (d) **SP1** in Ace using Hg^2+^, (e) **SP2** in MeOH using Cd^2+^, and (f) **SP2** in MeOH using Pb^2+^. Below each graph are photographs of the corresponding systems. The first vial contains the spiropyran derivative in 2 mL of the solvent with a 100 μL addition of pure water. In the subsequent vials, 100 μL of heavy metal ion solutions was added instead, resulting in final concentrations of 55.24, 11.05, 2.21, and 0.44 μmol L^–1^, respectively.

The photographs clearly show distinct visual changes compared to the control when the most concentrated stock solutions of the metal ions were added to all systems through either a noticeable color shift or, in the case of **SP2**, a complete loss of color. Notably, **SP1** exhibited visible changes even at lower mercury concentrations, with the most diluted solution also showing slight discoloration, indicating a high sensitivity to this metal ion. However, **SP1** was less sensitive to cadmium, showing noticeable changes only at the two highest concentrations in acetone and only at the highest concentration in propanol.

What is particularly interesting about these observations is that **SP1** exhibited distinct visual responses to cadmium and mercury in the same solvent (Ace). In the presence of mercury, the solution developed a noticeable yellowish hue, accompanied by a loss of the original color. In contrast, when exposed to cadmium, the color shifted from bluish-purple to dark pink. These distinguishable color changes suggest that **SP1**, in Ace, could potentially be used to selectively differentiate between cadmium and mercury by the naked eye. The combination of **SP2** and MeOH was the only system that potentially detected lead ions in solution. However, it exhibited a colorimetric response similar to that of cadmium, indicating reduced selectivity in this case.

### 3.4 Real Water Analysis

To assess the potential interference from other ions in the colorimetric detection of heavy metals, two real water samples were collected and spiked with the target ions used in the titration experiments. The first sample was obtained from the Portovelo, El Oro, region in Ecuador, an area potentially impacted by the contamination of tailings from extractive metallurgic processes. The second sample was collected from Quebrada Los Gringos, a stream impacted by acid rock drainage discharges from mining operations, in Torata, Santa Rosa, El Oro, Ecuador. Prior to spiking, both samples were analyzed to identify the presence of other elements that could interfere with the spiropyran-based detection of heavy metals. Notably, the sample from Portovelo contained elevated levels of copper, while the sample from Torata showed high concentrations of iron. These finding are consistent with the source of contamination in each case, tailings and acid rock drainages, respectively.^38^ A detailed elemental analysis is provided in Table SI 1. The samples were then spiked with heavy metal solutions to achieve final concentrations consistent with those used in the earlier titration experiments, and the photographs are shown in Figure 5.

**Figure 5:**
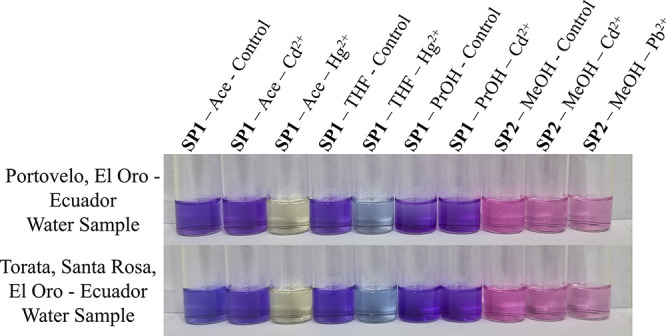
Photographs of the systems studied in quantitative experiments. Solutions were prepared with 2 mL of the indicated solvent containing spiropyran at a concentration of 0.025 mmol L^–1^. For the control vials, 100 μL aliquots of unaltered water samples were added. In the remaining vials, 100 μL of the same water samples spiked with heavy metals was added, resulting in a final metal ion concentration of 55.24 μmol L^–1^.

As shown in the photographs, the presence of various other ions in solution interfered with the colorimetric detection of Cd^2+^ and Pb^2+^, regardless of the solvent or spiropyran derivative used. In contrast, the response to Hg^2+^ remained consistent in real contaminated water samples when compared with the results obtained in Milli-Q water. This suggests that mercury ions can be reliably detected using **SP1** in both Ace and THF, even in the presence of competing ions commonly found in environmental samples.

Table 1 presents other studies for the detection of Cd^2+^, Pb^2+^, and Hg^2+^ heavy metal ions using spiropyran derivatives. As observed, different chemical modifications in the spiropyran skeleton have been proposed in order to improve the binding affinity and heavy metal ion selectivity. In addition, the capability of these ions’ detection varies not only due to the spiropyran derivative but also based on the physical-chemical analysis method employed (*e.g.*, absorption, emission, or naked eye color change). The **SP1** used in the present study was capable of detecting Hg^2+^ 0.44 μmol L^–1^ based on the naked eye color change, comparable to other spiropyran derivates for the same heavy metal ion using a similar approach (digital image analysis).^44^

**Table 1:**
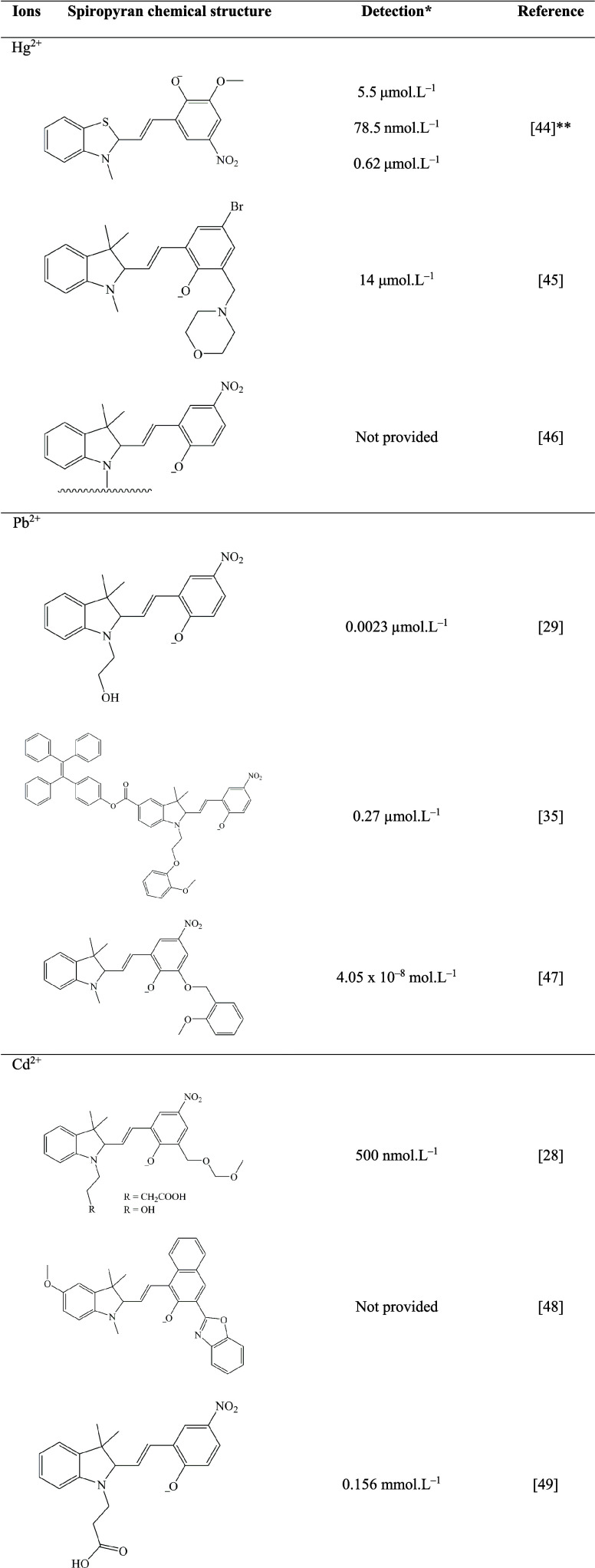
Spiropyran Derivatives Used for the Heavy Metal Ion Detection of Hg^2+^, Pb^2+^, and Cd^2+^ ^28,29,35,44–49^

* Units maintained as presented in the references.

** Based on the following methods: UV–vis, fluorescence spectroscopy, and digital image analysis.

## 4 Conclusions

Based on the obtained results, **SP1** and **SP2** exhibited solvent-dependent colorimetric responses that enable the selective detection of Hg^2+^ over Pb^2+^ and Cd^2+^ in solution. **SP3** did not exhibit any relevant spectral or visual change in any combination of metal-solvent that could be attributed to the interaction of the spiropyran and the studied ions. The emission spectra revealed solvent effects similar to the absorption studies, with all compounds exhibiting large Stokes shifts. Distinct absorbance shifts in **SP1** and **SP2** under specific solvent conditions were observed for these ions in four different solvents, highlighting the critical role of solvent polarity in tuning the sensor behavior. Pb^2+^ ions were detected with only a single system, **SP2** in MeOH, with low sensibility. Notably, Hg^2+^ ions induced a purple to yellow color change (with **SP1** in THF and Ace), which occurred even at low concentrations (0.44 μmol L^–1^) and in the presence of potential interferents, demonstrating high practical selectivity. Our study emphasizes the promise of solvent-tuned spiropyran probes for rapid, naked-eye detection of Hg^2+^ ions in the field, without the need for laboratory techniques or incorporation in complex materials. These advancements may contribute to the development and design of spiropyran-based probes for field-ready heavy metal monitoring.

## Supplementary Material



## Notes

Description of the spiropyran derivative (**SP1**, **SP2**, and **SP3**) synthesis, UV–vis spectra for heavy metal screening of **SP1**, **SP2**, and **SP3**, UV–vis spectra for **SP1**, **SP2**, and **SP3** during the dilution process, table of metal ion concentrations in real samples (PDF)
