# Supplementary material for: Exploiting Spiropyran Solvatochromism for Heavy Metal Ion Detection in Aqueous Solutions
Source: ACS Omega. 2025 Aug 6;10(32):36412–20. doi: 10.1021/acsomega.5c04821 (PMC12368694; doi:10.1021/acsomega.5c04821)
Supplement: Supplementary file 1 [file ao5c04821_si_001.pdf]

Supporting Information

Exploiting Spiropyran Solvatochromism for Heavy Metal Ion Detection in Aqueous Solutions

Nathália E. N. Mendonça [a], Carlos A. S. Leão [b], Frank Alexis [c], Valeria Ochoa-Herrera [d], Aracely Zambrano-Romero [d], Roberto S. Nobuyasu [b], Flávio B. Miguez [a], and Frederico B. De Sousa [a]**

[a]Laboratório de Sistemas Poliméricos e Supramoleculares (LSPS) – Instituto de Física e Química, Universidade Federal de Itajubá (UNIFEI), Itajubá, 37500-903, MG, Brazil.

[b]Laboratório de Fotofísica Molecular – Instituto de Física e Química, Universidade Federal de Itajubá (UNIFEI), Itajubá, 37500-903, MG, Brazil.

[c]Departamento de Ingeniería Química, Institute of Energies and Materials – Colegio de Ciencias e Ingenierías, Universidad San Francisco de Quito (USFQ), Quito,170901, Ecuador.

[d] Colegio de Ciencias e Ingenierías, AQUA-BIO Lab, Universidad San Francisco de Quito (USFQ), Quito,170901, Ecuador.

Corresponding authors:

Prof. Frederico B. De Sousa

Laboratório de Sistemas Poliméricos e Supramoleculares (LSPS) – Instituto de Física e Química, Universidade Federal de Itajubá (UNIFEI), Itajubá, 37500-903, MG, Brazil.

55 35 3629175

fredbsousa@gmail.com, fredbsousa@unifei.edu.br

*Flávio B. Miguez

Laboratório de Sistemas Poliméricos e Supramoleculares (LSPS) – Instituto de Física e Química, Universidade Federal de Itajubá (UNIFEI), Itajubá, 37500-903, MG, Brazil.
miguez.flavio@unifei.edu.br

Supplementary Information SI 1: Synthesis of spiropyran derivates

SP1 was synthesized following a combination of literature protocols [1,2]. A mixture of 2,3,3-trimethylindolenine (2.50 g, 15.7 mmol) and 3-iodopropanoic acid (3.16 g, 15.8 mmol) was heated under nitrogen at 100 °C for 3 h with constant stirring. The resulting purple solid was dissolved in water and washed with chloroform. After evaporating the water, l-(β,8-carboxyethyl)-2,3,3-trimethylindolenine iodide was obtained (90% yield) and used without further purification. This iodide (5.16 g, 14.3 mmol) was then reacted with 3-methoxy-5-nitrosalicylaldehyde (2.83 g, 14.3 mmol) in the presence of 4-methylpiperidine (1.42 g, 14.3 mmol) and 2-butanone (40 mL) under nitrogen reflux at 80 °C for 4 h with continuous stirring. The reaction mixture was then left refrigerated for 48 h, after which the precipitate was filtered and washed with cold acetone, yielding **SP1** as a solid (2.60 g, 44%).

SP2 was synthesized following a literature protocol with a single modification, replacing piperidine with 4-methylpiperidine. The synthesis began by heating a mixture of 2,3,3-trimethylindolenine (2.50 g, 15.7 mmol) and 3-iodopropanoic acid (3.16 g, 15.8 mmol) under nitrogen at 100 ºC for 3 h with constant stirring. The resulting purple solid was dissolved in water, washed with chloroform, and dried, yielding l-(β,8-carboxyethyl)-2,3,3-trimethylindolenine iodide (87%), which was used without purification. This iodide (4.95 g, 13.7 mmol) was then reacted with 2-hydroxy-5-nitrobenzaldehyde (2.30 g, 13.7 mmol) in the presence of 4-methylpiperidine (1.4 g, 14.1 mmol) and 2-butanone (40 mL) under nitrogen reflux at 80 ºC for 4 h. After standing refrigerated for 24 h, the precipitate was filtered, washed with cold methanol, and dried, affording **SP2** (3.46 g, 66%)

SP3 was synthesized following a literature protocol [1]. A solution of 1,3,3-trimethyl-2-methyleneindoline (1.76 g, 1.0 mmol) and 3-methoxy-5-nitrosalicylaldehyde (2.00 g, 1.0 mmol) in anhydrous ethanol (120 mL) was heated to reflux for 5 h. The solvent was then removed under reduced pressure, and the crude product was recrystallized from ethanol (30 mL). The resulting solid was filtered, washed with ethanol and water, and dried under high vacuum, yielding **SP3** as yellow-green crystals (3.03 g, 96%).

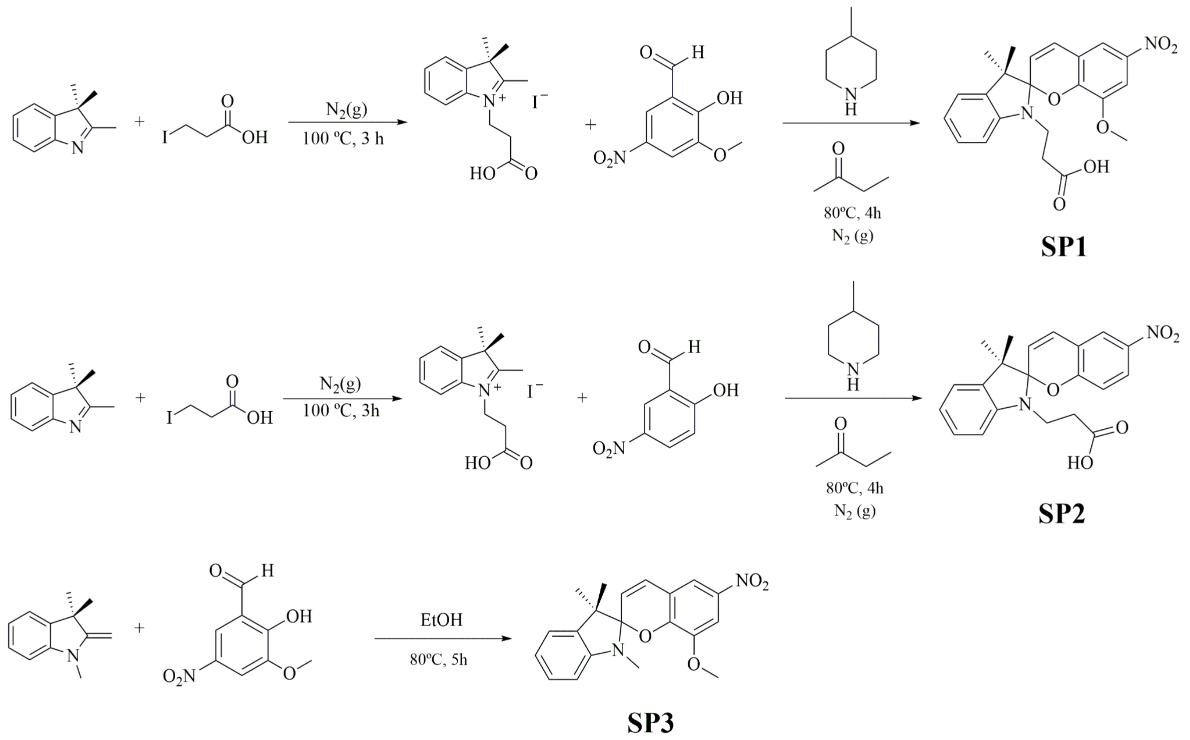

Figure SI 1. Synthesis of **SP1**, **SP2** and **SP3**.

Supplementary Information: Figure SI 2

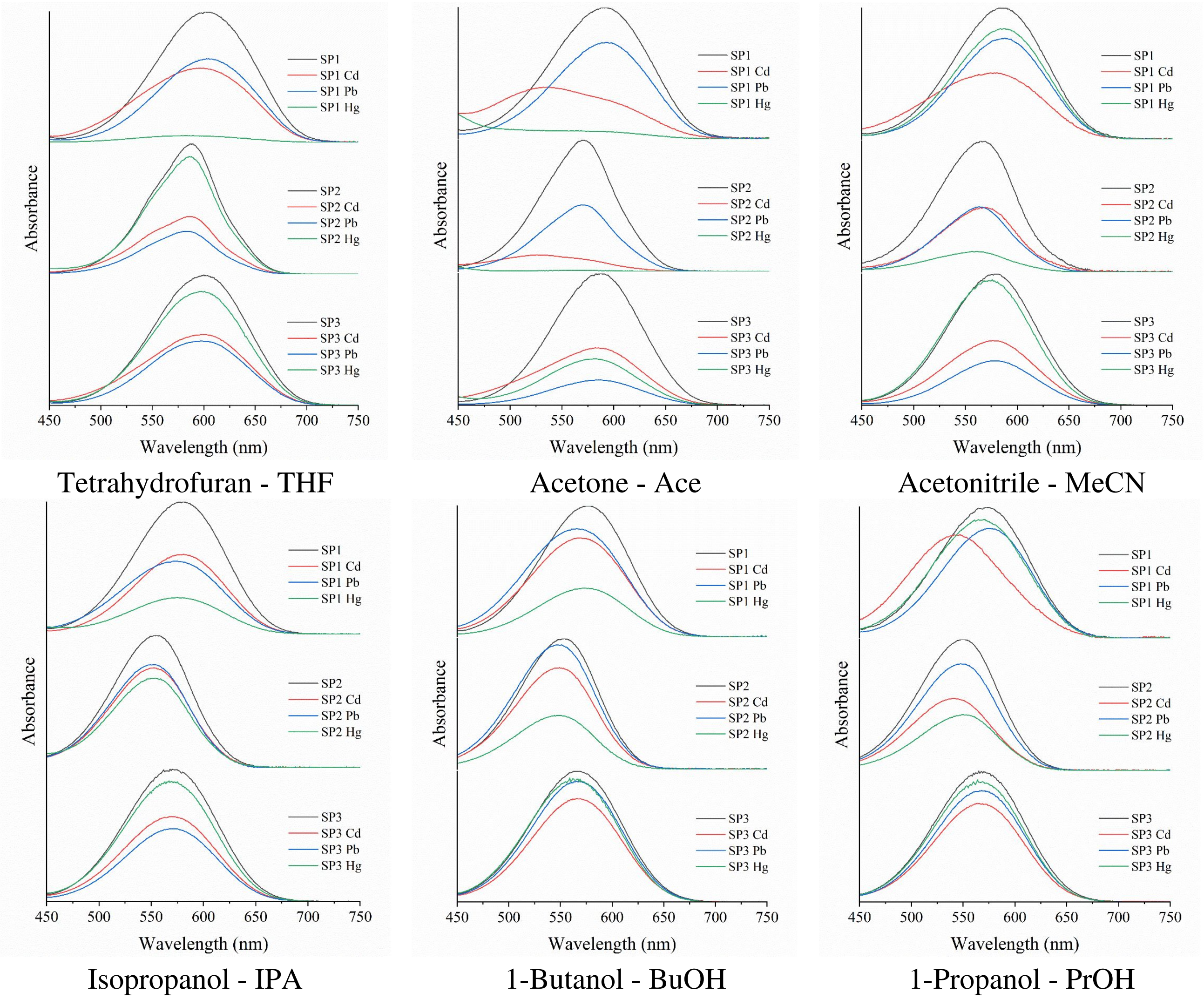

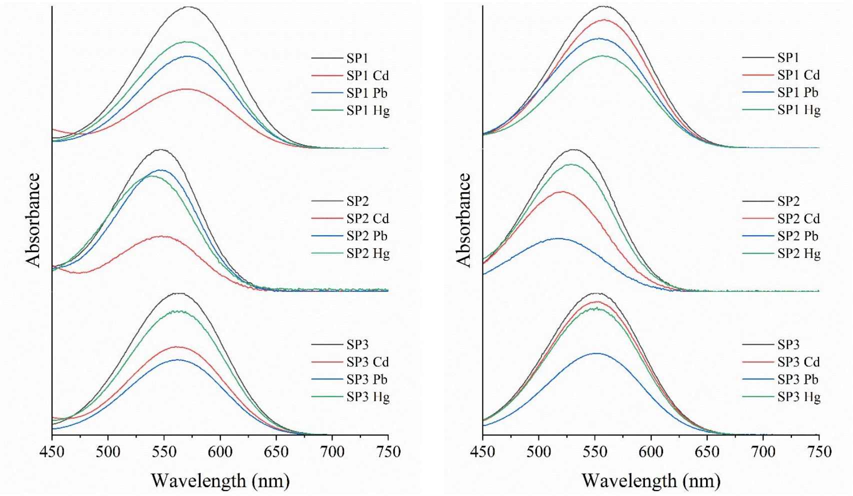

Ethanol - EtOH Methanol - MeOH

Figure SI 2: screening of the heavy metal ions using the electronic transition of spiropyran derivatives in different solvents.

Supplementary Information: Figure SI3

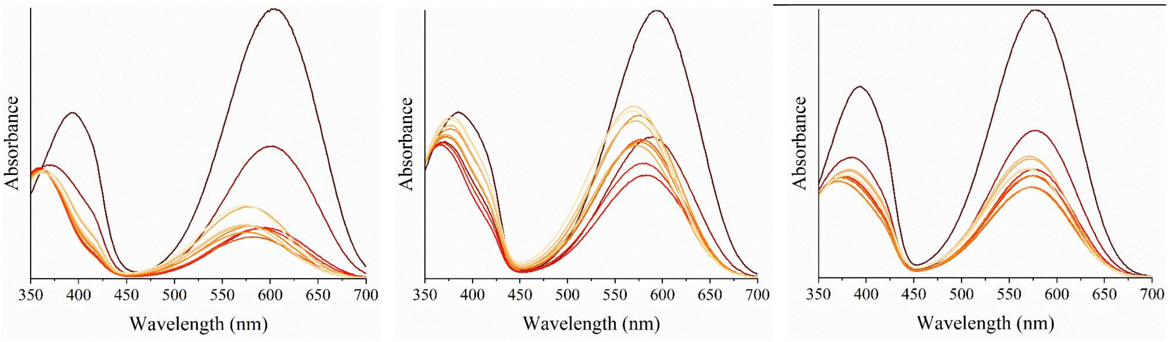

SP1 – THF **SP1** – Ace **SP1** – IPA

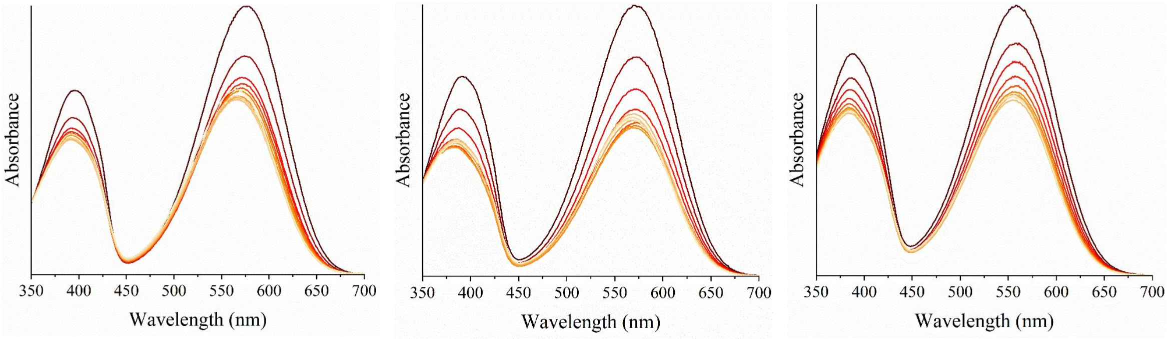

SP1 – PrOH **SP1** – EtOH **SP1** – MeOH

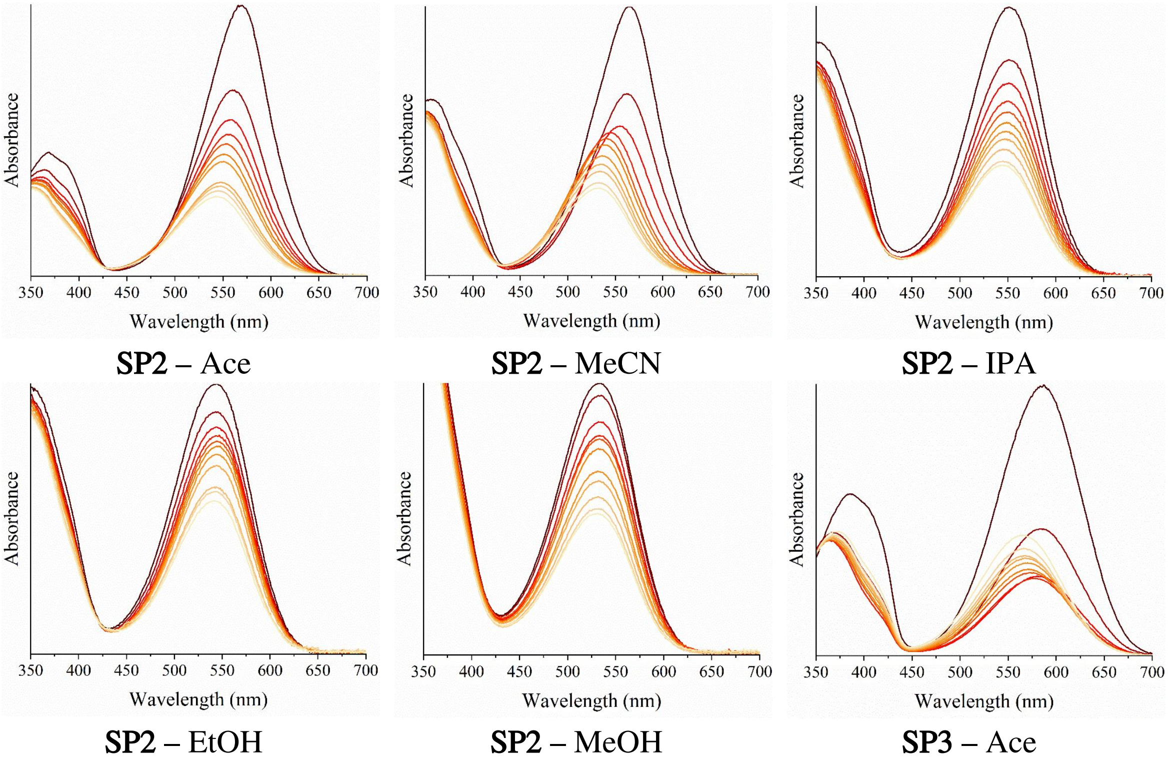

Figure SI 3: dilution process of spiropyran derivatives using Milli-Q® water.

Supplementary Information: Table SI 1

Table SI 1: Quantification of metal ions in real samples used for the heavy metal ions detection.

**Metals**	Sample from Portovelo, El Oro, Ecuador ($mg.L^{-1}$)	Sample from Quebrada Los Gringos, Torata, Santa Rosa, El Oro, Ecuador ($mg.L^{-1}$)
Barium, Ba	0.01	0.08
Copper, Cu	51.08	0.32
Chromium, Cr	<LOQ	<LOQ
Lead, Pb	0.1	0.04
Manganese, Mn	1.51	2.28
Molybdenum, Mo	0.09	<LOQ

Nickel, Ni	0.36	0.11
Zinc, Zn	0.18	2.00
Cobalt, Co	0.09	0.07
Cadmium, Cd	<LOQ	0.01
Arsenic, As	0.18	0.02
Iron, Fe	1.13	17.37
Lithium, Li	0.01	0.01
Selenium, Se	0.16	<LOQ
Antimony, Sb	0.18	0.15
Titanium, Ti	<LOQ	0.008
Strontium, Sr	0.21	0.60

**LOQ: Limit of quantification*

References:

[1] Feuerstein TJ, Müller R, Barner-Kowollik C, Roesky PW. Investigating the Photochemistry of Spiropyran Metal Complexes with Online LED-NMR. Inorg Chem 2019;58:15479–86. https://doi.org/10.1021/acs.inorgchem.9b02547.

[2] Fissi A, Pieroni O, Ruggeri G, Ciardelli F. Photoresponsive Polymers. Photomodulation of the Macromolecular Structure in Poly(L-lysine) Containing Spiropyran Units. Macromolecules 1995;28:302–9. https://doi.org/10.1021/ma00105a042.
